# Association of Serum Total Osteocalcin Concentrations With Endogenous Glucocorticoids and Insulin Sensitivity Markers in 12-Year-Old Children: A Cross-Sectional Study

**DOI:** 10.3389/fendo.2019.00798

**Published:** 2019-11-19

**Authors:** Satu Seppä, Sirpa Tenhola, Raimo Voutilainen

**Affiliations:** ^1^Department of Pediatrics, Kuopio University Hospital and University of Eastern Finland, Kuopio, Finland; ^2^Department of Pediatrics, Kymenlaakso Central Hospital, Kotka, Finland

**Keywords:** bone, cortisol, child, insulin, glucocorticoid, glucose metabolism, growth spurt, puberty

## Abstract

**Background:** Osteocalcin (OC) is an osteoblast-derived marker of bone turnover that has recently been linked to glucose metabolism, glucocorticoid action, and cardiovascular risk.

**Objective:** We determined whether serum total OC (tOC) is associated with cardiometabolic factors, such as insulin sensitivity (IS) markers and endogenous glucocorticoids in 12-year-old children. In addition, we assessed whether low birth weight or exposure to maternal preeclampsia affect tOC concentrations.

**Methods:** In this cross-sectional study, 192 children (109 girls) were studied at 12 years of age. Seventy of them had been born small (SGA), 78 appropriate for gestational age (AGA), and 44 from preeclamptic pregnancies (PRE) as AGA. Blood pressure was measured, and fasting blood samples were collected for markers of glucose metabolism, osteoblast, adipocyte, and adrenocortical function. IS was estimated by Quantitative Insulin Sensitivity Check Index (QUICKI). Free cortisol index (FCI) was calculated as serum cortisol/corticosteroid binding globulin.

**Results:** The highest tOC concentrations were detected in midpubertal children (Tanner B/G stage 3). The children in the highest tOC quartile (*n* = 48) had lower body mass index (BMI), waist-to-height ratio, diastolic blood pressure, leptin, cortisol/cortisone ratio and FCI, and higher insulin-like growth factor I (IGF-I), IGF-binding protein-1 (IGFBP-1), IGFBP-3, and alkaline phosphatase (ALP) than those in the lower tOC quartiles (*p* < 0.02 for all). QUICKI was similar in these subgroups. In logistic regression analysis, pubertal developmental stages 2 and 3, high ALP, IGF-I, and low FCI and BMI (*p* < 0.02 for all) were associated independently with higher tOC. The means of serum tOC and IS markers were similar in the SGA, AGA, and PRE subgroups.

**Conclusions:** In both sexes, the highest tOC levels were detected in midpubertal children reflecting the fast pubertal growth phase. Higher tOC levels were associated with lower BMI and FCI, whereas no association was found with IS. Birth weight or exposure to preeclampsia had no effect on tOC concentrations.

## Introduction

Osteocalcin (OC) is a commonly used marker of bone turnover (1, 2), which has recently been linked to glucose and energy metabolism (2). OC is a non-collagenous polypeptide existing abundantly in the mineralized bone matrix. After being synthetized by osteoblasts, it undergoes vitamin K-dependent carboxylation. The γ-carboxylation of OC increases its affinity for hydroxyapatite crystals; thus, most of the produced OC is embedded in the bone matrix (1–3). A small amount of OC abides undercarboxylated (ucOC) and is secreted into the circulation (1, 2). Therefore, both carboxylated and ucOC forms exist in the circulation (3). UcOC has been suggested to act as a hormone linking bone to the mediators of glucose homeostasis (4, 5). In animal models, circulating ucOC has been shown to regulate whole-body energy metabolism by increasing insulin sensitivity in peripheral tissues, enhancing insulin secretion from pancreatic beta cells and upregulating adiponectin secretion from fat cells (4–6).

In human studies, circulating total OC (tOC) concentrations were positively associated with markers of insulin sensitivity (IS) (7), and negatively with plasma glucose (3, 7, 8), insulin resistance (3, 7, 9), body mass index (BMI) (8–10), fat mass (8), and waist circumference (10). Adults with type 2 diabetes (3, 7, 8, 11–13) or the metabolic syndrome (7) had lower tOC concentrations compared with healthy subjects. In cross-sectional studies, tOC was inversely associated with the risk of type 2 diabetes, the metabolic syndrome (7) and cardiovascular mortality (3). Lower tOC levels were also found in overweight or obese adults (3, 10) and children (9). Furthermore, ucOC was inversely associated with measures of glycemia and diabetes risk even after adjustments for tOC in large epidemiological studies conducted in older men (3), and positively associated with beta cell function in children with prediabetes (14). However, some longitudinal studies in elderly or middle-aged adults showed no association between tOC or ucOC and glycemic control (3).

Serum tOC concentrations vary by age and sex (15–17). Throughout childhood, circulating tOC levels are higher than the adult reference range. The concentrations of tOC peak with the fast pubertal growth phase at Tanner stages 2–3 of breast development in girls and at Tanner stages 2–3 of genital development in boys. TOC concentrations decrease to adult level at the end of statural growth (17).

Glucocorticoids are known to induce insulin resistance, hyperglycemia and hyperlipidemia via multiple pathways. Glucocorticoid action in bone impairs osteoblast function and thus decreases OC synthesis, which in turn contributes to insulin resistance according to animal studies (18, 19). In patients with Cushing's syndrome (CS), bone formation is greatly suppressed. Sereg et al. found that subjects with overt CS had the lowest serum tOC concentrations when compared to subjects with subclinical CS, hormonally inactive adrenal adenomas or those without hypothalamo-pituitary-adrenal axis disturbances. Serum tOC concentrations correlated inversely with serum and salivary cortisol levels in overt CS. Thus, it was suggested that serum OC may sensitively reflect endogenous hypercortisolism (20).

The purpose of the present study was to evaluate, whether serum tOC concentrations associate with cardiometabolic factors, such as markers of IS, endogenous glucocorticoids, and blood pressure (BP) in children aged 12 years. Furthermore, we wanted to determine, whether tOC concentrations differ in children born small (SGA) or appropriate (AGA) for gestational age, or from preeclamptic pregnancies (PRE). Based on previous reports, we hypothesized that serum tOC would associate inversely with unfavorable cardiometabolic factors and that SGA and PRE children could have lower tOC concentrations than AGA-born ones, as low birth weight and maternal preeclampsia prompt independently later metabolic and cardiovascular disturbances (21, 22).

## Materials and Methods

### Study Design and Subjects

The design of this study was cross-sectional. The study population consisted of a cohort of 192 12-year-old children, who were recruited in the study investigating metabolic and cardiovascular consequences of either being born SGA or after PRE. Of this cohort 109 (57%) were females, 70 (36%) were born SGA, and 44 (23%) were born after PRE as AGA. 16 (23%) of the SGA subjects (8% of the cohort) were born after PRE. The median of the gestational ages was 38.0 weeks (range 28-42). The extremely preterm children born before the week 28 of gestation were excluded from the study. None of the participating children was exposed to exogenous glucocorticoids prenatally. All children were born at Kuopio University Hospital during a 22-months period between 1984 and 1986, and they were evaluated between 1996 and 1998. The study protocol was approved by the Research Ethics Committee of Kuopio University Hospital. Informed written consent was obtained from the child and the parents in accordance with the Declaration of Helsinki.

### Variables of the Study

#### Early Life Variables

Preeclampsia was defined as the development of hypertension and proteinuria (>300 mg of urinary protein in 24 h) after 20 weeks of gestation (23). Hypertension was defined as BP >140/90 mmHg or a rise of ≥30/15 mmHg from the baseline level confirmed by two measurements at least 6 h apart. Full-term indicates babies born at or after week 37 and before the 42nd week of gestation, and preterm indicates babies born before the 37th week of gestation (calculated from the beginning of the last menstruation). SGA was defined as birth weight and/or length more than 2 SD scores below the respective mean for the gestational age and sex. AGA was defined as birth weight and birth length equal to or above −2 SD scores and equal to or below +2 SD scores of the respective mean for the gestational age and sex (24). The variables were collected from the medical reports of the mothers and study subjects.

#### Pubertal and Anthropometric Variables

The perinatal data and anthropometric measures at 12 years age have been described previously for the SGA (25, 26) and PRE children (27, 28). Pubertal development was classified as Tanner stages according to breast development (B) in girls and genital development (G) in boys. BMI (calculated as weight in kilograms divided by height in meters squared) and sex- and adult age -adjusted BMI [BMIadj; corresponding to the BMI values at the age of 18 years (29)] were calculated. Waist-to-height ratio (WHtR) was calculated by dividing waist circumference (in centimeters) by height (in centimeters). Perinatal characteristics, anthropometric measures, and pubertal development at the age of 12 years in the whole study population are presented in Table 1.

**Table 1:** Anthropometric characteristics of the study population at birth and at the age of 12 years.

**Variable**	**All (*n* = 192)**	**Girls (*n* = 109)**	**Boys (*n* = 83)**	p^*^	p^**^
**AT BIRTH**					
Gestational age (wk)•	37.5 (37.0, 38.0)	37.9 (37.3, 38.6)	37.0 (36.3, 37.8)	0.069	
Weight (g)	2,769 (2,662, 2,877)	2,736 (2,586, 2,886)	2,813 (2,659, 2,967)	0.482	
Weight (SDS)	−1.14 (−1.33, −0.95)	−1.27 (−1.55, −1.00)	−0.97 (−1.23, −0.71)	0.111	
Length (cm)	47.3 (46.7, 47.8)	47.0 (46.3, 47.8)	47.6 (46.8, 48.4)	0.260	
Length (SDS)	−0.88 (−1.09, −0.67)	−1.02 (−1.31, −0.74)	−0.69 (−1.00, −0.38)	0.117	
**AT THE AGE OF 12 YEARS**					
Age (year)•	12.25 (12.23, 12.27)	12.25 (12.22, 12.29)	12.25 (12.21, 12.28)	0.868	
Weight (kg)•	43.00 (41.60, 44.44)	43.14 (41.26, 45.09)	42.81 (40.70, 45.03)	0.816	
BMIadj (kg/m^2^)•	21.15 (20.63, 21.68)	20.65 (20.02, 21.31)	21.81 (20.96, 22.70)	**0.028**	**0.001**[Table-fn TN3] 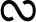
Height (cm)	153.2 (152.1, 154.2)	153.9 (152.4, 155.4)	152.2 (150.6, 153.8)	0.119	
Height (SDS)	0.26 (0.12, 0.41)	0.16 (−0.04, 0.36)	0.40 (0.18, 0.61)	0.116	
WHtR	0.43 (0.42, 0.44)	0.42 (0.41, 0.43)	0.44 (0.43, 0.45)	**0.013**	**0.012**[Table-fn TN3] 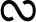
Pubertal development^¶^ [early/late stage (*n* (%))]	107/85 (56/44%)	38/71 (35/65%)	69/14 (83/17%)	**<0.001**^∞^	
Pubertal development				**<0.001**^∞^	
Tanner B/G stage 1 (*n* (%))	40 (21%)	14 (13%)	26 (31%)		
Tanner B/G stage 2 (*n* (%))	67 (35%)	24 (22%)	43 (52%)		
Tanner B/G stage 3 (*n* (%))	50 (26%)	38 (35%)	12 (15%)		
Tanner B/G stage 4 (*n* (%))	27 (14%)	25 (23%)	2 (2%)		
Tanner B/G stage 5 (*n* (%))	8 (4%)	8 (7%)	0		

*Means (95% confidence intervals) are presented, geometric means (95% confidence intervals) (•) are presented for the variables with skew distributions. Significant p-values are bolded*.

* *Independent-Samples T-test for the differences between the girls and boys*.

** *Comparison by Analysis of covariance (ANCOVA) adjusted for BMIadj and pubertal developmental stage (G/B 1-5)*.

*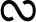 Comparison by ANCOVA adjusted for pubertal developmental stage (G/B 1-5)*.

∞ *Chi-Square test for the differences between the girls and boys*.

¶ *Early stage of puberty: the breast or genital scores 1-2, late stage of puberty: the breast or genital scores 3-5.BMIadj, BMI adjusted for sex and adult age; SDS, Standard deviation score; WHtR, waist-to-height ratio*.

#### Biochemical Variables

Blood samples were taken in the morning, between 9 AM and 10 AM, after an overnight fast. An intravenous cannula was placed in the antecubital vein for blood sampling. After the child had rested for 1 h in a recumbent position, blood samples were drawn through the cannula. Blood glucose concentrations were measured after sampling, plasma and serum specimens were immediately frozen and stored at −70°C until analyzed. The biochemical, hormonal, and adipocytokine analyses were performed later using previously unthawed serum aliquots.

Serum tOC concentrations were measured by enzyme-linked immunosorbent assay (ELISA) (N-MID Osteocalcin ELISA, Nordic Bioscience Diagnostics A/S, Herlev, Denmark). The intra- and interassay coefficients of variation (CV) for tOC were <2.2 and <5.1%, respectively, as reported by the manufacturer. Serum alkaline phosphatase (ALP) concentrations were analyzed by enzymatic photometric test (Konelab 20XT Clinical Chemistry Analyzer, Thermo Fisher Scientific, Vantaa, Finland), and the respective CVs were 4.0% and <5.7%. Serum calcium (Ca) concentrations were measured by Photometric colorimetric test, Arsenazo III (Konelab 20 XT, Clinical Chemistry Analyzer, Thermo Fisher Scientific) with its intra- and interassay CVs 1.7% and <2.0, respectively. Serum insulin concentrations were determined by radioimmunoassay (RIA) (Phadeseph Insulin RIA, Pharmacia & Upjohn Diagnostics AB, Uppsala, Sweden). The intra- and interassay CVs were 5.3 and 7.6%, respectively. Blood glucose concentrations were analyzed by a glucose oxidase method (Enzyme Electrode, Nova Biomedical, Waltham, MA), and the respective CVs were 3 and 5%. Serum insulin-like growth factor I (IGF-I) concentrations were analyzed by ELISA (DSL-10-5600 ACTIVE IGF-I ELISA; Diagnostic Systems Laboratories, Inc., Webster, TX) as described previously (30). The intra-assay CV for IGF-I was 6.5%, and the interassay CV was 6.4%, as reported by the manufacturer. Serum IGF-binding protein-1 (IGFBP-1) and IGFBP-3 concentrations were analyzed by ELISA (DSL-10-7800 ACTIVE Total IGFBP-1 ELISA, DSL-10-6600 ACTIVE IGFBP-3 ELISA, both from Diagnostic Systems Laboratories). The intra- and interassay CVs for IGFBP-1 were 2.5 and 6.8%, and for IGFBP-3 7.3 and 8.2%, respectively. Serum sex hormone binding globulin (SHBG) was measured by the AutoDELFIA SHBG time-resolved fluoroimmunoassay method (Perkin Elmer Life Sciences Wallac, Turku, Finland). The intra- and interassay CVs were 4.0 and 2.6%, respectively. Serum leptin and adiponectin concentrations were analyzed by ELISA (Quantikine DLP00; Quantikine DRP300, both from R&D Systems, Inc., Minneapolis, MN) with their intra- and interassay CVs 3.2 and 3.5% (leptin), and <4.7 and <6.9% (adiponectin), respectively. Serum cortisol and cortisone concentrations were analyzed by liquid chromatography tandem mass spectrometry as previously described (31). Serum corticosteroid binding globulin (CBG) was measured by RIA (CBG-RIA-100, Biosource Europe, Nivelles, Belgium), as described previously (32). Free cortisol index (FCI) was calculated as serum cortisol (nmol/L)/CBG (mg/L) (33). QUICKI (Quantitative Insulin Sensitivity Check Index) was calculated as 1/[log (fasting insulin, μU/mL) + log (fasting glucose, mg/dL)] (34).

#### Statistical Analyses

Data were analyzed using the statistical program SPSS for Macintosh, version 24.0 (SPSS, IBM Corp., Armonk, NY). All continuous variables were examined for normality with the Kolmogorov-Smirnov test. Skewed data were either logarithmically or square root -transformed before testing. Independent samples *T*-test, univariate analysis of variance (ANOVA) or analysis of covariance (ANCOVA) were used for comparisons between different groups. Sidak-correction was used for *post hoc* tests. Partial correlation coefficients were calculated. Most of the analyses were adjusted for BMIadj and pubertal stage in order to eliminate their confounding effect on the results. Logistic regression analysis was used to explore independent associating factors with serum tOC. In our model, the dependent variable was serum tOC concentration (the highest quartile) and the independent variables were pubertal stages B/G 1-5, BMIadj, ALP, IGF-I, IGFBP-1, FCI, sex, birth weight (SDS), and maternal preeclamptic history.

A *p* < 0.05 was accepted as significant in all analyses.

## Results

### Anthropometric Characteristics and Pubertal Development

The anthropometric characteristics and pubertal development of the study subjects are presented in Table 1. The mean age of the children at the examination was 12.25 years, and 56.8% of them were females. The means of birth weight and length were similar between the girls and boys. At 12 years of age, the boys had greater BMIadj and WHtR than the girls, whereas pubertal development was more advanced in the girls than boys (Table 1). Anthropometric characteristics and pubertal development of the SGA, AGA and PRE-AGA children were described recently (35).

### Serum tOC, Glucocorticoid Parameters and Markers of IS

The mean concentrations of serum tOC, ALP, and IGFBP-3 did not differ between the sexes, whereas the means of serum IGF-I and leptin were higher in the girls than boys. At 12 years of age, the girls had lower means of QUICKI and IGFBP-1 than the boys. The serum concentrations of cortisol, cortisone, the cortisol/cortisone ratio and FCI were similar between the sexes. The mean of CBG was lower in the girls; however, after adjustment for BMIadj and pubertal developmental stage, the difference turned non-significant (Table 2). No differences were found in tOC, ALP, QUICKI, cortisol, and FCI between the AGA, SGA, and PRE-AGA subgroups (data not shown). The cortisol/cortisone ratio was higher in the PRE children (including both AGA and SGA subjects) compared with the non-PRE ones (2.99 vs. 2.72, *p* = 0.030 by ANCOVA adjusted for sex, pubertal developmental stage, and BMIadj).

**Table 2:** Biochemical parameters in the study population (girls and boys separately).

**Variable**	**All (*n* = 192)**	**Girls (*n* = 109)**	**Boys (*n* = 83)**	p^*^	p^***^
S-Total osteocalcin (ng/mL) •	126.4 (120.4, 132.8)	131.3 (122.7, 140.5)	120.3 (112.3, 129.0)	0.069	0.212
S-ALP (U/L) •	272.8 (261.3, 284.8) *n* = 191	265.3 (249.9, 281.7) *n* = 108	282.8 (265.9, 300.9)	0.162	0.399
S-Ca (mmol/L)	2.58 (2.55, 2.61) *n* = 191	2.57 (2.54, 2.61) *n* = 108	2.59 (2.55, 2.63)	0.507	0.150
S-IGF-I (ng/mL)	314.3 (296.3, 332.4)	360.5 (338.2, 382.7)	253.8 (228.9, 278.6)	**<0.001**	**<0.001**
S-IGFBP-3 (ng/mL)	4,576 (4,366, 4,787)	4,734 (4,459, 5,010)	4,369 (4,041, 4,696)	0.071	0.482
S-Insulin (mU/L) •	9.4 (8.9, 9.9)	10.3 (9.5, 11.1)	8.3 (7.7, 9.0)	**<0.001**	**<0.001**
B-Glucose (mmol/L)	4.3 (4.3, 4.4)	4.3 (4.2, 4.3)	4.4 (4.3, 4.5)	**0.009**	**0.025**
QUICKI	0.351 (0.348, 0.354)	0.347 (0.342, 0.352)	0.356 (0.351, 0.361)	**0.007**	**0.005**
S-IGFBP-1 (ng/mL) •	56.4 (51.8, 61.4)	51.6 (45.9, 58.0)	63.4 (56.0, 71.7)	**0.017**	**0.007**
S-SHBG (nmol/L)	73.5 (68.9, 78.2)	69.6 (63.7, 75.5)	78.7 (71.3, 86.2)	0.060	**0.032**
S-Leptin (ng/mL) •	8.6 (7.3, 10.1)	11.2 (9.2, 13.6)	6.1 (4.7, 7.8)	**<** **0.001**	**<0.001**
S-Adn (mg/L) •	9.4 (8.7, 10.2)	9.8 (8.9, 10.8)	8.9 (7.7, 10.2)	0.375	0.150
S-Cortisol (nmol/L)•	205.8 (194.4, 217.8)	201.9 (187.1, 217.8)	211.0 (193.5, 230.0)	0.501	0.627
S-Cortisone (nmol/L)	80.6 (77.0, 84.2)	80.4 (75.9, 84.9)	80.9 (75.0, 86.8)	0.888	0.458
Cortisol/cortisone -ratio •	2.68 (2.57, 2.80)	2.63 (2.48, 2.79)	2.75 (2.59, 2.92)	0.382	0.973
S-CBG (μg/mL)	64.8 (62.8, 66.7)	62.3 (60.0, 64.6)	68.0 (64.8, 71.2)	**0.005**	0.096
FCI •	3.24 (3.06, 3.44)	3.30 (3.06, 3.56)	3.18 (2.89, 3.49)	0.605	0.906
24-h systolic BP (mmHg)	117 (116, 118) (*n* = 180)	116 (115, 118) (*n* = 100)	117 (116, 119) (*n* = 80)	0.354^**^	0.272^****^
24-h diastolic BP (mmHg)	68 (68, 69) (*n* = 180)	68 (67, 69) (*n* = 100)	69 (68, 70) (*n* = 80)	0.380^**^	0.239^****^

*The means (95% confidence intervals) are presented, geometric means (95% confidence intervals) (•) are presented for the variables with skew distributions. Significant p-values are bolded*.

* *Independent-Samples T-test for the differences between the girls and boys*.

** *Comparison by Analysis of covariance (ANCOVA) adjusted for BMIadj and height (SDS)*.

*** *Comparison by ANCOVA adjusted for BMIadj and pubertal developmental stage (G/B 1-5)*.

**** *Comparison by ANCOVA adjusted for BMIadj, pubertal developmental stage (G/B 1-5) and height (SDS). Adn, adiponectin; ALP, alkaline phosphatase; BMIadj, BMI adjusted for sex and adult age; BP; blood pressure; Ca, calcium; CBG, corticosteroid binding globulin; FCI, free cortisol index; IGF-I, insulin-like growth factor 1; IGFBP-1, insulin-like growth factor-binding protein-1; IGFBP-3; insulin-like growth factor-binding protein-3; QUICKI, Quantitative Insulin Check Index; SDS, standard deviation score; SHBG, sex hormone-binding globulin*.

### Serum tOC in Relation to Anthropometric Measures, IS Markers, and Glucocorticoid Parameters

In the whole study population, serum tOC had a negative correlation with BMIadj and WHtR. When the sexes were analyzed separately, these correlations were significant only in the girls (Table 3). Serum tOC had no correlation with blood glucose, serum insulin, QUICKI and height SD scores. Serum tOC correlated positively with ALP and Ca in the whole study population and in the girls. Furthermore, positive correlations between serum tOC and IGF-I were observed in the whole study population and in sex-specific analyses, whereas the correlation between serum tOC and IGFBP-3 was significant in the whole study population and in the boys (Table 3). In the whole study population, serum tOC associated negatively with SHBG, leptin and adiponectin (Table 3). Finally, in the whole study population and in the girls, serum tOC correlated negatively with the cortisol/cortisone ratio. In the girls, serum tOC correlated inversely with cortisol and FCI (Table 3).

**Table 3:** Correlation analyses between serum total osteocalcin and anthropometric and biochemical parameters.

**Variable**	**All** ***n* = 192**		**Girls** ***n* = 109**		**Boys** ***n* = 83**	
	***r***	***p***	***r***	***p***	***r***	***p***
BMIadj^*^	−0.244	**0.001**	−0.329	**<0.001**	−0.097	0.386
WHtR^*^	−0.237	**0.001**	−0.271	**0.005**	−0.092	0.410
Height (SDS)	0.142	0.051	0.132	0.174	0.112	0.319
S–ALP	0.340 (*n* = 191)	**<0.001**	0.397 (*n* = 108)	**<0.001**	0.006	0.958
S-Ca	0.161 (*n* = 191)	**0.027**	0.386 (*n* = 108)	**<0.001**	−0.178	0.113
S-IGF-I	0.266	**<0.001**	0.213	**0.028**	0.254	**0.022**
S-IGFBP-3	0.214	**0.003**	0.183	0.060	0.231	**0.038**
S-Insulin	0.061	0.408	0.097	0.319	0.020	0.857
B-Glucose	0.010	0.891	0.092	0.348	−0.106	0.347
QUICKI	−0.079	0.283	−0.132	0.176	−0.012	0.916
S-IGFBP-1	0.109	0.136	0.080	0.416	0.199	0.075
S-SHBG	−0.241	**0.001**	−0.347	**<0.001**	−0.073	0.519
S-Leptin	−0.174	**0.017**	−0.081	0.407	−0.190	0.090
S-Adiponectin	−0.164	**0.024**	−0.128	0.188	−0.135	0.229
S-Cortisol	−0.138	0.058	−0.231	**0.017**	0.007	0.947
S-Cortisone	0.031	0.672	0.001	0.995	0.021	0.854
Cortisol/cortisone -ratio	−0.254	**<0.001**	−0.313	**0.001**	−0.079	0.484
S-CBG	−0.074	0.313	−0.063	0.518	0.079	0.485
FCI	−0.100	0.172	−0.210	**0.030**	−0.029	0.799

*Partial correlation analyses (adjusted for BMIadj and pubertal developmental stage) between serum tOC and anthropometric and biochemical parameters in the whole study population and in the girls and boys separately. In the whole study population, the analyses are adjusted also for sex. Significant p-values are bolded*.

* *Adjusted for pubertal developmental stage*.

*ALP, alkaline phosphatase; BMIadj, BMI adjusted for sex and adult age; Ca, calcium; CBG, corticosteroid binding globulin; FCI, free cortisol index; IGF-I, insulin-like growth factor 1; IGFBP-1, insulin-like growth factor-binding protein-1; IGFBP-3; insulin-like growth factor-binding protein-3; QUICKI, Quantitative Insulin Check Index; SDS, standard deviation score; SHBG, sex hormone-binding globulin; WHtR, waist-to-height ratio*.

### Serum OC, ALP, IGF-I, IGFBP-3, and Insulin Across Pubertal Stages

**Figure 1:**
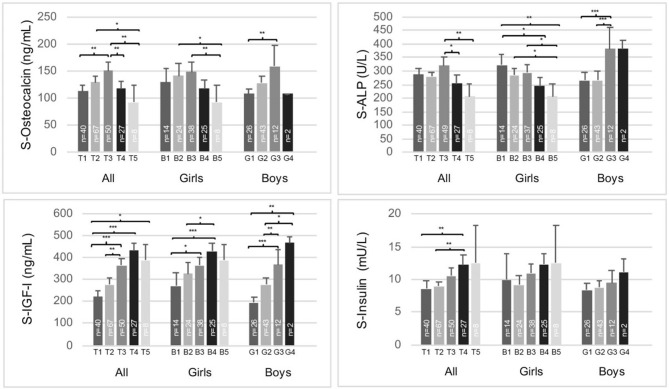
Serum total osteocalcin, alkaline phosphatase (ALP), IGF-I, and insulin concentrations according to Tanner stages B/G 1-5 in the whole study population (girls and boys separately). The means (95% CI) are presented. Analysis of covariance (adjusted for gender in the whole study population) were performed, Sidak-adjusted *p*-values are presented. *p < 0.05, **p < 0.01, ***p < 0.001.

In both sexes, the highest tOC concentrations were found in midpubertal children, in Tanner stage B/G 3 (Figure 1). ALP concentrations were highest in Tanner stage G3 in the boys and B1 in the girls (Figure 1). In the girls, serum IGF-I peaked in Tanner stage B4 (Figure 1), whereas IGFBP-3 was highest in Tanner stage B3 without a significant difference to other stages (data not shown). In the boys, serum IGF-I (Figure 1) and IGFBP-3 increased during puberty achieving the highest levels in the Tanner stage G4; however, the IGFBP-3 levels were not significantly different between the Tanner stages (data not shown). Moreover, none of the male subjects had yet completed pubertal development (reached Tanner stage G5). The prepubertal (Tanner stage 1) girls had higher serum tOC and ALP concentrations than the prepubertal boys (Figure 1, *p* = 0.030, *p* = 0.015, respectively, by Independent-Samples *T*-test). Furthermore, IGF-I was higher in the girls than boys in Tanner stages 1 and 2 (Figure 1, *p* = 0.007 and *p* = 0.006, respectively, by Independent-Samples *T*-test). However, the boys had higher ALP values compared with the girls in Tanner stages 3 (Figure 1, *p* = 0.003) and 4 (Figure 1, *p* = 0.044, by Independent-Samples *T*-test). In the whole study population, serum insulin concentrations increased during puberty; however, the increases were not statistically significant, when the sexes were analyzed separately (Figure 1).

### Factors Associating With High Serum tOC Concentrations

The children in the highest tOC quartile were leaner in terms of BMIadj and WHtR, and they had lower leptin concentrations than the children in the lower tOC quartiles. No difference was found in height (SD scores), but the progression of pubertal development differed between these subgroups. In the highest tOC quartile, 44% of the subjects were in midpuberty (Tanner stage B/G 3), whereas in the three lower tOC quartiles the corresponding percentage was 20% (Table 4). The means of QUICKI, blood glucose and serum insulin concentrations were similar between the highest and three lower quartiles, whereas IGF-I, IGFBP-1, IGFBP-3, ALP, and Ca concentrations were higher in the children with the highest tOC levels than in those with lower tOC concentrations (Table 4). The observed differences in IGFBP-1 and IGFBP-3 concentrations between the tOC quartiles were diminished after adjustment for confounding factors BMIadj and pubertal developmental stage (Table 4). Furthermore, SHBG levels were lower in the highest tOC quartile compared with those in the lower quartiles in BMIadj-adjusted analyses (Table 4). Serum cortisol and cortisone concentrations were similar between the highest and the lower tOC quartiles. However, the mean of the cortisol/cortisone ratio was lower in the highest tOC quartile than in the three lower quartiles (Table 4) independently of gender, pubertal developmental stage and BMIadj. After adjustments for confounding factors, the subjects in the highest tOC quartile had lower FCI than those with lower tOC concentrations (Table 4). The difference remained even after adjustment for birth weight (SD scores) and maternal PRE history. The children in the highest tOC quartile had lower 24-h systolic and diastolic BP means compared with the children in the lower tOC quartiles. However, after additional adjustment for height (SD scores), the difference in 24-h systolic BP between the groups was attenuated.

**Table 4:** Comparison between the highest and three lower osteocalcin quartiles.

	**S-total osteocalcin,** **the highest quartile** **(*n* = 48)**	**S-total osteocalcin,** **three lower quartiles** **(*n* = 144)**	p^*^	p^**^
Gender (*n* = f/m)	33/15	76/68	0.053^¶^	
Birth weight (SDS)	−1.37 (−1.70, −1.05)	−1.07 (−1.30, −0.83)	0.124	0.361
Birth length (SDS)	−1.15 (−1.52, −0.79)	−0.79 (−1.04, −0.54)	0.134	0.383
Pubertal development at 12 years age			**0.002**^¶^	
Tanner B/G stage 1 (*n* = f/m) [%]	4 (3/1) [8 (6/2)%]	36 (11/25) [25 (8/17)%]	**0.014**^¶^	
Tanner B/G stage 2 (*n* = f/m) [%]	19 (10/9) [40 (21/19)%]	48 (14/34) [33 (10/23)%]	0.431^¶^	
Tanner B/G stage 3 (*n* = f/m) [%]	21 (16/5) [44 (33/11)%]	29 (22/7) [20 (15/5)%]	**0.001**^¶^	
Tanner B/G stage 4 (*n* = f/m) [%]	4 (4/0) [8 (8/0)%]	23 (21/2) [16 (15/1)%]	0.187^¶^	
Tanner B/G stage 5 (*n* = f/m) [%]	0	8 (8/0) [6 (6/0)%]	0.095^**¶**^	
BMIadj (kg/m^2^) •	19.8 (19.0, 20.7)	21.6 (21.0, 22.3)	**0.001**	
WHtR	0.42 (0.40, 0.43)	0.44 (0.43, 0.45)	**0.008**	0.438
Height (SDS) at 12 years age	0.23 (−0.02, 0.48)	0.28 (0.10, 0.45)	0.763	0.347
S–Leptin (ng/mL) •	5.7 (4.3, 7.4)	9.8 (8.1, 11.9)	**0.001**	0.085
S-Adiponectin (mg/L) •	8.5 (7.3, 10.0)	9.7 (8.8, 10.7)	0.123	0.073
S-Insulin (mU/L) •	8.9 (8.1, 9.8)	9.5 (8.9, 10.2)	0.223	0.802
B-Glucose (mmol/L)	4.33 (4.25, 4.42)	4.33 (4.27, 4.39)	0.909	0.552
QUICKI	0.353 (0.348, 0.359)	0.350 (0.346, 0.354)	0.356	0.902
S-Osteocalcin (ng/mL) •	198.5 (187.1, 210.5)	108.8 (104.8, 113.0)	**<0.001**	**<0.001**
S-ALP (U/L) •	315.2 (292.3, 340.0)	259.9 (247.3, 273.0) (*n* = 143)	**<0.001**	**0.001**
S-Ca (mmol/L)	2.63 (2.57, 2.68)	2.56 (2.53, 2.59) (*n* = 143)	**0.036**	0.079
S-IGF-I (ng/mL)	349.5 (319.5, 379.6)	302.6 (280.8, 324.4)	**0.004**	**0.019**
S-IGFBP-3 (ng/mL)	4,993 (4,621, 5,365)	4,438 (4,188, 4,688)	**0.017**	0.089
S-IGFBP-1 (ng/mL) •	69.0 (61.3, 82.8)	52.7 (47.5, 58.6)	**0.006**	0.185
S-SHBG (nmol/L)	68.9 (60.8, 77.7)	75.1 (69.5, 80.7)	0.253	**0.003**
S-Cortisol (nmol/L)•	191.0 (169.6, 215.0)	211.0 (197.7, 225.0)	0.169	0.057
S-Cortisone (nmol/L)	81.5 (75.1, 87.9)	80.3 (76.0, 84.6)	0.788	0.745
Cortisol/cortisone -ratio •	2.42 (2.22, 2.65)	2.77 (2.64, 2.91)	**0.010**	**0.010**
S-CBG	64.6 (60.9, 68.3)	64.8 (62.6, 67.1)	0.968	0.481
FCI •	3.01 (2.70, 3.35)	3.33 (3.10, 3.57)	0.130	**0.025**
24-h systolic BP (mmHg)	114 (112, 116) (*n* = 43)	118 (116, 119) (*n* = 137)	**0.012**	0.127^***^
24-h diastolic BP (mmHg)	67 (65, 68) *n* = 43	69 (68, 70) (*n* = 137)	**0.010**	0.020^***^

*The means (95% confidence interval) are presented, geometric means (95% confidence intervals) (•) are presented for the variables with skew distributions. Significant p-values are bolded*.

¶ *Chi-Square test*.

* *Independent-Samples T-test*.

** *Analysis of covariance (ANCOVA) adjusted for gender, pubertal developmental stage (G/B 1-5) and BMIadj*.

*** *ANCOVA adjusted for gender, pubertal developmental stage (G/B 1-5), BMIadj and height (SDS)*.

*ALP, alkaline phosphatase; BMIadj, BMI adjusted for sex and adult age; BP, blood pressure; Ca, calcium; CBG, corticosteroid binding globulin; FCI, free cortisol index; IGF-I, insulin-like growth factor 1; IGFBP-1, insulin-like growth factor-binding protein-1; IGFBP-3; insulin-like growth factor-binding protein-3; QUICKI, Quantitative Insulin Check Index; SDS, standard deviation score; SHBG, sex hormone-binding globulin; WHtR, waist-to-height ratio*.

In a binary logistic regression model adjusted for sex, birth weight (SD scores) and maternal PRE history, Tanner stages B/G 2-3, high serum IGF-I and ALP concentrations, and low FCI and BMIadj associated independently with higher tOC concentrations (Table 5).

**Table 5:** Factors associating with high total osteocalcin (the highest osteocalcin quartile) in the whole study population.

**Covariate**	**Regression coefficient**	**Significance**	**Odds ratio (OR)**	**95% confidence interval for OR**
Tanner pubertal stage 2 (vs. Tanner pubertal stage 1)	1.996	0.008	7.36	1.67–32.43
Tanner pubertal stage 3 (vs. Tanner pubertal stage 1)	2.010	0.009	7.46	1.64–33.91
Low BMIadj (kg/m^2^)	0.255	0.005	1.30	1.08–1.54
High S–IGF-I (10 ng/mL)	0.050	0.017	1.05	1.01–1.10
High S-ALP (10 U/L)	0.082	0.002	1.09	1.03–1.14
Low S-FCI (0.1 units)	0.034	0.018	1.03	1.01–1.06
High S-IGFBP-1 (10 ng/mL)	0.106	0.138	1.11	0.97–1.28

*Results of the binary logistic regression analysis (n = 189) adjusted for sex, birth weight (SDS) and maternal preeclamptic pregnancy history; variation explained by the model 41.9%. ALP, alkaline phosphatase; BMIadj, BMI adjusted for sex and adult age; FCI, free cortisol index; IGF-I, insulin-like growth factor 1; IGFBP-1, insulin-like growth factor-binding protein-1*.

## Discussion

In this study, serum tOC was not associated with IS estimated by QUICKI in 12-year-old children. The highest tOC concentrations were detected in midpubertal children with high IGF-I, IGFBP-3, and ALP concentrations. Furthermore, higher tOC associated with lower FCI and cortisol/cortisone ratio. In binary logistic regression analysis, Tanner stages B/G 2-3, high IGF-I and ALP concentrations, and low FCI and BMIadj associated independently with a high tOC value. Being born SGA or prenatal exposure to maternal preeclampsia was not associated with tOC concentrations.

Cross-sectional human studies including subjects with variable age, sex and adiposity distribution have shown inverse associations between tOC or ucOC and adiposity, measures of glycemia and insulin resistance (3, 7–9, 36). However, many studies have found no association between tOC or ucOC and indices of glycemia cross-sectionally or over time (3, 37–39), or significant associations have been demonstrated only in subjects with metabolic dysregulation, such as impaired glucose tolerance or prediabetes (3, 14). Consequently, the association between tOC and ucOC and glucose metabolism has been suggested to be age-dependent and minor under normal glucose tolerance or BMI (37). On the contrary, circulating tOC has been reported to associate with IS and insulin secretion in lean adults (40). In longitudinal observational studies with elderly adults, higher serum tOC was associated with a lower rise in fasting plasma glucose, and increased serum tOC concentrations were associated with an increase in insulin secretion, whereas an increase in ucOC was associated with enhanced IS (3). Inconclusive findings have led to speculations that ucOC and carboxylated OC could have divergent roles in the human glucose-insulin axis (38). However, numerous confounding factors such as age and sex differences, vitamin K status, physical activity, BMI, IS, and renal function may explain the observed inconsistencies in different studies.

So far, relatively few studies on the relation between OC forms and IS have been performed in children or adolescents, and the results have been inconsistent. Reinehr et al. (9) reported in 11-year-old children a significant negative relationship between tOC and HOMA-IR. In another study with 5 to 9-year-old lean children, a higher ratio of ucOC to carboxylated OC associated with higher HOMA-β, but not with HOMA-IR (36). In 7–11-year-old prepubertal overweight children, carboxylated OC was associated with IS in both normal-glucose and prediabetic children, whereas ucOC was associated with beta cell function only in prediabetic subjects (14). A study of Polgreen et al. (10) performed in young (17–22 years old) adults showed only a non-significant borderline relation between tOC and insulin resistance estimated by euglycemic hyperinsulinemic clamp; no independent relationship between ucOC and insulin resistance was detected. Redondo et al. found an inverse correlation between ucOC and hemoglobin A1c shortly after diagnosis of pediatric diabetes (41). Furthermore, Jürimäe et al. reported a positive correlation between tOC and HOMA-IR in 11 to 12-years old normal-weight boys (42), whereas other studies have found no relationship in ucOC or tOC and markers of glucose metabolism in children or adolescents (43, 44). A study of Tubic et al. in 2 to 9-year-old children reported no correlation between OC forms and measured metabolic parameters (38). In accordance with these, we found no association between tOC and glucose homeostasis. The inconsistencies between the findings of different studies may be explained by the different methods used to assess OC, by the age of the study subjects or by different selection criteria of the study cohorts.

We found a negative correlation between tOC and BMIadj and WHtR in accordance with most previous studies in children or young adults (9, 10, 42–44). However, Tubic et al. (38) reported that only carboxylated OC was decreased in prepubertal overweight compared with normal-weight children, whereas a couple of studies have found no relationship between tOC concentrations and indicators of body weight (45, 46).

In young adults, serum tOC was inversely associated with systolic BP and a cardiovascular risk factor cluster score (10). In another study with prepubertal children at risk for cardiovascular disease, ucOC was positively related to cardiovascular risk markers (47), whereas in a recent meta-analysis serum tOC had no evident association with vascular calcification or atherosclerosis (48). In our study, 24-h systolic and diastolic BP means were significantly lower in the subjects in the highest tOC quartile; however, further adjustment for height (SD scores) attenuated the association between tOC and 24-h systolic BP.

The interaction between adipose and bone tissue has been speculated to be mediated partly by leptin and adiponectin (2). Experimental studies have shown that ucOC regulates IS by increasing adiponectin release from adipocytes (4). In contrast, leptin inhibits OC production by osteoblasts (2). Even in human studies, bone and glucose metabolism has been postulated to be connected through a complex pathway involving tOC, adiponectin and leptin (49). A positive association between serum carboxylated OC or tOC and adiponectin has been reported in adults (10, 13, 40), while the associations between tOC and insulin secretion, IS and glucose tolerance were independent of plasma adiponectin levels (13). Previous reports in children and adolescents (9, 42, 44, 45), and non-diabetic women (37) have reported no association between serum tOC and adiponectin, whereas we found a negative correlation when adjusted for BMIadj and pubertal developmental stage. In our study, tOC correlated negatively with leptin as reported previously (8, 9, 42, 44), although some studies have not found such an association in children or young adults (37, 39, 45).

The concentrations of circulating bone formation markers IGF-I, ALP, and tOC peak during the pubertal growth spurt and decline during late puberty (17, 45, 50–52). Thus, tOC levels are at their highest during the fast pubertal growth phase (17, 45, 50, 51, 53). TOC levels were previously described to rise at an earlier pubertal stage in females than males (47). We found the highest tOC concentrations in midpuberty in both sexes, and no difference was found in the mean tOC concentrations between girls and boys after the prepubertal phase. In most studies, boys are reported to have higher tOC concentrations than girls from midpuberty onwards (9, 45, 52, 53). In accordance with us, Yilmaz et al. (54) reported tOC concentrations to peak at Tanner stage 3; however, they observed no significant change in tOC concentrations across pubertal stages. The differences between the findings could be due to differences in methodology, or the selected cohorts. In our study, the mean serum IGF-I concentrations increased with pubertal stages in parallel with those of tOC until midpuberty; after that tOC levels started to decrease while IGF-I remained high as described by others (53). Furthermore, in our whole study population and in the girls, serum tOC correlated positively with ALP. However, no association was found between serum ALP and tOC in boys even after adjustments for BMIadj and pubertal developmental stage. This suggests that other factors, such as gonadal-skeletal axis is relevant in pubertal skeletal growth and metabolism as shown previously in experimental studies and in pubertal boys (42, 55, 56).

In mice, excessive glucocorticoids impair osteoblast function and lead to decreased circulating tOC and ucOC levels. Restoration of OC levels by gene transfer experiments prevented glucocorticoid-induced changes in energy metabolism and body composition (18). In humans, exogenous or endogenous glucocorticoid excess associates with adverse clinical features, such as osteoporosis, accumulation of visceral fat and insulin resistance, or diabetes. However, the significance of bone in glucocorticoid-induced changes in energy metabolism and body composition is unclear in humans (19). FCI, which eliminates the effect of CBG variation in cortisol values, correlates well with free serum cortisol considered the biologically active hormone (33). We showed that FCI was lower in the subjects of the highest tOC quartile compared with those of lower quartiles even after appropriate adjustments. This suggests that even individual physiological variation in serum cortisol may be reflected in circulating tOC levels. The high sensitivity of serum tOC concentrations to glucocorticoids was previously observed also in children receiving inhaled steroids for treatment of asthma (57).

Low birth weight and maternal preeclampsia predispose to later cardiometabolic diseases (21, 22). Altered activation of the hypothalamo-pituitary-adrenal axis has been proposed to play a role in intrauterine growth restriction (IUGR) (58). The cortisol/cortisone ratio has been used as an indirect measure of the enzyme activity of 11β-hydroxysteroid dehydrogenase type 2 (11β-HSD2) (59, 60), which converts cortisol to biologically inactive cortisone. In humans, reduced placental 11β-HSD2 gene expression has been reported in IUGR and PREs (61, 62). In the present cohort, tOC concentrations were similar in the SGA, AGA, and PRE children. However, the children born from PRE had a higher serum cortisol/cortisone ratio than those without exposure to maternal PRE.

The strengths of this study include detailed anthropometric data of the study population at birth and at 12 years of age, a careful examination of pubertal development and a large quantity of biochemical measurements. However, also potential limitations exist. The cross-sectional nature of this study allows evaluation of associations but not causality. The study age of 12 years is demanding due to the variable timing in pubertal development and transient hyperinsulinemia and insulin resistance occurring during midpuberty. We attempted to exclude the possible influence of these by adjusting the analyses for the pubertal developmental stage. Furthermore, the one-time measurement of IS could be a potential limitation in this study. Although fasting insulin alone or in combination with fasting glucose are not optimal measures for assessing individual IS, they are considered appropriate in studies with well-defined cohorts (63). QUICKI, which was used as a surrogate marker of IS in our study, correlates quite well with the glucose clamp method (64). Unfortunately, we had no data on the frequency and intensity of exercise and dietary habits, which could affect individual IS. Finally, we have no measurements of carboxylated and ucOC forms separately. UcOC and tOC correlate strongly; thus, tOC can still be considered a reliable bone turnover marker associating with parameters of energy metabolism (56).

In conclusion, at 12 years of age the highest tOC levels were observed in midpubertal children with high IGF-I, IGFBP-3 and ALP concentrations reflecting the fast pubertal growth phase. IS, being born SGA or prenatal exposure to maternal preeclampsia were not associated with tOC concentrations. Thus, tOC seems not to be associated with IS in normoglycemic children. However, higher tOC levels were associated with lower FCI indicating lower physiological glucocorticoid activity. The inverse association between tOC and FCI suggests that even the physiological variation in circulating endogenous glucocorticoid levels may influence osteoblast activity and circulating tOC concentrations.

## Data Availability Statement

The datasets generated for this study are available on request to the corresponding author.

## Ethics Statement

The studies involving human participants were reviewed and approved by Research Ethics Committee of Kuopio University Hospital Puijonlaaksontie 2, P.O. Box 100, FI-70029 Kuopio, Finland Written informed consent to participate in this study was provided by the participants' legal guardian/next of kin.

## Author Contributions

RV and ST designed the study. ST and SS performed collection and handling of the data. SS analyzed the data and wrote the manuscript with contributions of all authors. All authors discussed the data and accepted the final draft.

### Conflict of Interest

The authors declare that the research was conducted in the absence of any commercial or financial relationships that could be construed as a potential conflict of interest.

## Abbreviations

AGA: appropriate for gestational age
ALP: alkaline phosphatase
ANCOVA: analysis of covariance
ANOVA: analysis of variance
B: breast development
BMI: body mass index
BMIadj: BMI adjusted for sex and adult age
BP: blood pressure
Ca: calcium
CBG: corticosteroid binding globulin
CS: Cushing's syndrome
CV: coefficient(s) of variation
ELISA: enzyme-linked immunosorbent assay
G: genital development
HOMA-IR: Homeostasis Model Assessment for Insulin Resistance
IGF-I: insulin-like growth factor I
IGFBP-1: IGF-binding protein-1
IGFBP-3: IGF-binding protein-3
IS: insulin sensitivity
IUGR: intrauterine growth restriction
OC: osteocalcin
PRE: preeclamptic pregnancy
QUICKI: Quantitative Insulin Sensitivity Check Index
RIA: radioimmunoassay
SGA: small for gestational age
tOC: total osteocalcin
ucOC: undercarboxylated osteocalcin
WHtR: waist-to-height ratio
11β-HSD2: 11β-hydroxysteroid dehydrogenase type 2.
